# Value of metalloproteinases in predicting COPD in heavy urban smokers

**DOI:** 10.1186/s12931-020-01496-9

**Published:** 2020-09-02

**Authors:** Jun-Chieh J. Tsay, Yingjie Hu, Judith D. Goldberg, Bin Wang, Soumya Vijayalekshmy, Ting-An Yie, Katrina Bantis, Daniel H. Sterman, William N. Rom

**Affiliations:** 1 William N. Rom Environmental Lung Disease Laboratory, Division of Pulmonary, Critical Care, and Sleep Medicine, Department of Medicine, and Department of Environmental Medicine, New York University School of Medicine, New York, NY USA; 2 Division of Biostatistics, Department of Population Health and Department of Environmental Medicine, NYU School of Medicine, New York, NY USA

## Abstract

**Background:**

Emphysema in asymptomatic heavy smokers can be detected during CT-scan screening for lung cancer. Metalloproteinases (MMPs) have been found to play a role in the pathogenesis of chronic obstructive pulmonary disease and to possibly serve as biomarkers for emphysema.

**Methods:**

The NYU Lung Cancer Biomarker Center enrolled study subjects over 50 years of age with lung cancer risk factors from January 1, 2010, to December 31, 2015. These subjects received chest multi-detector computed tomography, spirometry, and provided serum for immunoassays for metalloproteinases (MMP) -1, -2, -7, -9, -10 and tissue inhibitor of metalloproteinases (TIMP) -1 and -2.

**Results:**

Three hundred sixteen study subjects were enrolled. Of the 222 patients who met the inclusion criteria, 46% had emphysema. Smokers with emphysema had increased pack-years of smoking compared to smokers without emphysema (51 ± 24 pack-years (mean ± sd) versus 37 ± 20; *p* < 0.0001). Smokers with emphysema also had lower FEV_1_/FVC percent compared to smokers without emphysema (68 ± 11 (mean ± sd) versus 75 ± 8; *p* < 0.0001). Increased age and pack-years of smoking were associated with increased odds of emphysema. None of the metalloproteinases or tissue inhibitors of metalloproteinases were useful to predict the presence of emphysema in smokers.

**Conclusion:**

Emphysema was detected by CT in almost half of heavy urban smokers. Serum MMP levels provided minimal additional information to improve the detection of mild emphysema among smokers given their clinical characteristics (age, pack-years, and FEV_1_/FVC ratio).

## Introduction

In the United States, the prevalence of chronic obstructive pulmonary disease (COPD) is estimated to be 24 million individuals, of which half remains undiagnosed, and it is the third leading cause of death worldwide [1]. Detection of emphysema may act as a catalyst for patients to quit smoking and subsequently prevent worsening lung function. While the U.S. Preventive Services Task Force recommends against screening adults for COPD using spirometry [2, 3], the respiratory societies do recommend spirometry for adults with respiratory symptoms, especially dyspnea [4]. Among the > 20,000 randomly selected participants in a large population based survey, the prevalence of COPD was 8.2%, however only 6.5% of these diagnosed subjects had ever been examined with spirometry [5]. Lung cancer screening can be useful to detect emphysema in asymptomatic heavy smokers, [73% sensitivity and 88% specificity based on the NELSON Trials [6]]. However, there will still be a cohort of asymptomatic smokers who will remain undiagnosed. The Multi-Ethnic Study of Atherosclerosis (MESA) study showed that smokers have an increased mortality if emphysema was present on CT scans [7], therefore it is important to detect subjects with asymptomatic emphysema.

Serum biomarkers can be useful to detect smokers with emphysema. Researchers have been studying the role of matrix metalloproteinases (MMPs) in COPD pathogenesis. D’Armiento and colleagues [8] reported that MMPs and tissue inhibitors of MMPs (TIMP) did not predict progression to emphysema. They found elevations of MMP levels (MMP-1, -9, -12, TIMP -1) in the bronchoalveolar lavage (BAL) of the emphysematous lung. The aim of this study is to evaluate the utility of serum MMP levels in the identification of patients with asymptomatic emphysema. We hypothesized that in asymptomatic smokers (or those with exposure to second-hand smoke), serum MMP and TIMP biomarkers could improve predicting the presence of emphysema.

## Methods

### Study cohort

**Fig. 1:**
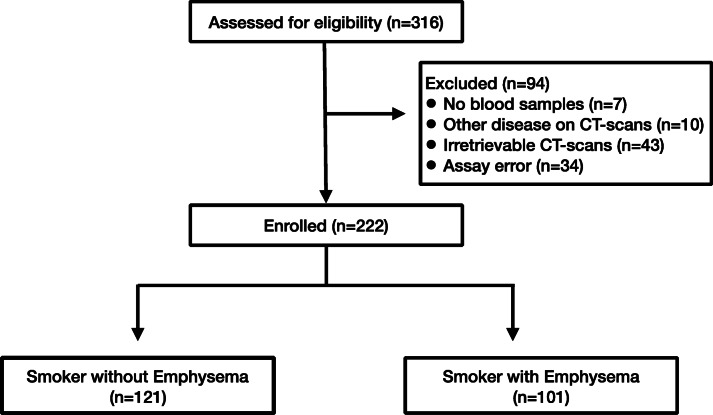
CONSORT Diagram: Screening to Enrollment in Emphysema/No Emphysema Groups

We enrolled 316 study subjects from January 1, 2010, to December 31, 2015. Subjects were excluded if CT-scans indicated other disease findings, were irretrievable from outside hospitals or no blood samples were available (See Fig. 1). Two hundred and fifty six of these remaining subjects were > 50 years of age, most with smoking histories of over 20 pack-years who consented to participate in the lung cancer screening protocol of the NYU Lung Cancer Biomarker Center. Among those 256 subjects, 34 samples were excluded due to technical errors that occurred during assay processing. The distribution of the demographics and clinical characteristics of these 34 subjects were similar to the remaining subjects in the dataset. After excluding these patients, samples from the remaining 222 patients were analyzed. This study was approved by the NYU Institutional Review Board. Each participant completed a low-dose computerized tomography (CT) of the chest, a baseline demographic questionnaire, and a peripheral blood draw. We utilized the American Thoracic Society (ATS) respiratory questionnaire which included questions on demographic characteristics, tobacco use, occupation, occupational exposures, alcohol use, family history, and medical history (Supporting Document). Spirometry was performed by a trained technician according to ATS standards.

### CT chest radiograph

CT-scans, the gold standard for detecting emphysema, utilized 64 multi-detector scanners obtaining 5 mm–thick images; images were reconstructed every 5 mm using 1 mm collimation. This technique allowed simultaneous prospective reconstruction of contiguous 1 mm images for high-resolution detailed analysis. CT-scans were read by a thoracic radiologist and confirmed by two additional pulmonologists for the presence of emphysema using a qualitative assessment.

### MMP measurements

The Luminex bead multiplex assay (Austin, Texas, USA) was utilized for measuring serum MMPs -1, -2, -7, -9, -10 and TIMPs -1 and -2, as per manufacturer’s instruction. The patients were classified into two groups: smokers with emphysema (*n* = 101), and smokers without emphysema (*n* = 121).

### Statistical methods

In addition to detecting emphysema we sought to evaluate whether a panel of biomarkers (MMPs -1, -2, -7, -9, and -10, and TIMPs -1 and -2) could distinguish emphysema from no emphysema among heavy smokers. Distributions of patients and disease characteristics including clinical features, smoking history, pulmonary function, and other variables were compared using frequency distributions and proportions using Fisher’s exact tests for categorical variables.

Seventy-eight of the 222 eligible subjects (35%) had undetectable MMP-10 measurements below the limits of detection. Therefore, we used binary classes (detectable, undetectable: detectable if value ≥27 pg/ml and undetectable if value < 27 pg/ml) to explore the potential utility of this marker.

Biomarker levels were measured in two batches because limited laboratory personnel and assay kits were available to pull the sequentially identified samples for thaw and aliquot. Batch 1 had 44 smokers with emphysema and 68 without emphysema, while in Batch 2 there were 57 with emphysema and 53 non-emphysema. The analyses of the batch effects using Fisher’s exact test suggested that the distributions of subjects with and without emphysema did not differ significantly between batches (56% of batch 1 and 44% of batch 2 had no emphysema (Supplement Table 1, *p* = 0.08). Nonparametric Wilcoxon rank-sum tests were used to evaluate differences in the distributions of individual biomarkers between the two batches with respect to emphysema and non-emphysema separately. The results indicated that all biomarkers had significantly different distributions by batch with the exception of MMP-1 (Supplement Table 2). Batch effects were adjusted with a log_2_ transformation to each biomarker to remove skewness and the biomarker levels were standardized within each batch. After standardization within batches, the Wilcoxon rank sum test indicated that the distributions of the biomarkers of emphysema (and of smokers) did not differ significantly between the two batches (Supplement Table 2).

We first used stepwise multiple logistic regression with bidirectional variable selection to distinguish smokers with emphysema from those without emphysema, including only their clinical characteristics (Model 1: Age, BMI, pack-years, FEV1/FVC ratio). Next, we used the same regression method and included both clinical variables and biomarkers (MMP1, MMP2, MMP7, MMP9, TIMP1 and TIMP2) to obtain Model 2. An additional model that included only the set of biomarkers (MMPs/TIMPs) was also evaluated (Model 3). Receiving Operating Characteristic (ROC) curves were plotted from these logistic models; Areas Under the Curve (AUC) and Akaike Information Criterion (AIC) [9] for the resulting models were calculated under these three scenarios for comparison of these models. The Youden Index [10] was used to identify the optimal cut points for the models and estimates of sensitivity and specificity of each model provided at the cut point. All data analyses were performed with R version 3.4.3, MASS package version 7.3-48, and pROC package 1.15.0.

## Results

We recruited 316 individuals; 222 were included in these analyses (Fig. 1). Distributions of baseline characteristics of the 222 subjects are shown in Table 1. There were 101 smokers with emphysema (46%) and 121 smokers without emphysema (54%). The smokers with emphysema were older (65 ± 7 years) than those without emphysema (61 ± 7 years; *p* < 0.0001). There were no differences in gender (*p* = 0.42). Further, there were no differences in BMI (emphysema 28 ± 5, non-emphysema 27 ± 5; *p* = 0.24). There were more current smokers in the emphysema group (emphysema 49% versus non-emphysema 31%; *p* = 0.01). There was an increase in pack-years among smokers with emphysema (51 ± 24 pack-year) compared to smokers without emphysema (37 ± 20 pack-years; *p* < 0 .0001).

**Table 1:** Clinical Characteristics by Emphysema Status in Smokers in Batch 1 and Batch 2 (222 subjects)

	Smokers without Emphysema		Smokers with Emphysema		*P* value
	Mean ± SD or %	N	Mean ± SD or %	N	
Age (years)^†^	60.8 ± 7.1	121	64.8 ± 7.0	101	**< 0.0001**
Height (cm)^†^	169.1 ± 10.0	120	170.3 ± 9.2	101	0.51
Weight (kg)^†^	80.9 ± 17.8	120	79.4 ± 18.7	101	0.50
BMI^†^	28.2 ± 5.3	120	27.2 ± 5.3	101	0.24
Start smoke age^†^	16.9 ± 4.0	121	16.6 ± 4.5	101	0.80
Cigarettes Daily^†^	24.0 ± 11.0	121	26.3 ± 11.1	101	0.11
Smoke Duration Years^†^	31.7 ± 11.5	121	39.6 ± 10.8	101	**< 0.0001**
Pack Years^†^	37.2 ± 20.4	121	50.9 ± 23.8	101	**< 0.0001**
FEV_1_/FVC^†^	75.5 ± 7.9	111	67.5 ± 10.6	95	**< 0.0001**
Gender		121		102	
Female	61 (50%)		45 (45%)		0.42
Male	60 (50%)		57 (55%)		
Race		121		102	0.06
African American	4 (3%)		9 (9%)		
Asian	0 (0%)		1 (1%)		
Caucasian	110 (91%)		90 (89%)		
Indian	1 (1%)		0 (0%)		
Other	6 (5%)		1 (1%)		
Cough		121		102	**0.04**
Yes	20 (17%)		29 (29%)		
No	101 (83%)		73 (71%)		
Current Smoker		121		102	**0.01**
Yes	38 (31%)		49 (49%)		
No	83 (69%)		52 (51%)		
Phlegm		120		101	0.11
Yes	8 (7%)		14 (14%)		
No	112 (93%)		86 (86%)		
MRC Score		121		101	0.12
0	87 (72%)		63 (62%)		
1	12 (10%)		23 (23%)		
2	8 (7%)		5 (5%)		
3	2 (2%)		3 (3%)		
4	6 (5%)		5 (5%)		
5	6 (5%)		2 (2%)		
MMP10		121		101	0.40
Detectable	75 (62%)		69 (68%)		
Undetectable	46 (38%)		32 (32%)		

^†^ *P* values from nonparametric Wilcoxon Rank-Sum rank sum test (2-sided). All other *P* values based on

Fisher’s Exact Test

*P* values ≤0.05 bolded

### Clinical characteristics

The FEV_1_/FVC ratio was lower in those with radiographic emphysema (emphysema 68 ± 11%(SD) versus non-emphysema 76 ± 8%(SD); *p* < 0.0001). A higher percentage of emphysema subjects had symptoms of cough (emphysema 29%, non-emphysema 17%; *p* = 0.04) and reports of phlegm (emphysema 14%, non-emphysema 7%; *p* = 0.11) than those without emphysema. Next, three stepwise multiple logistic models were conducted to explore the extent to which MMP biomarkers could help identify emphysema among smokers. Table 2 shows the results of Model 1, a stepwise multiple logistic regression with bidirectional elimination using clinical characteristics including age, smoking duration, pack-years, cough and FEV1/FVC ratio. With the exception of smoking duration, the other 4 variables were all highly associated with emphysema among smokers.

**Table 2:** Odds Ratios and 95% Confidence Interval (CI) for Emphysema Based on Stepwise Multiple Logistic Regression models with Clinical Characteristics Only. (222 subjects)

Predictors	OR	95% CI
Age	1.07	(1.02, 1.12)
Pack-Years	1.02	(1.01, 1.04)
FEV_1_/FVC	0.93	(0.89, 0.96)
Cough	1.91	(0.87, 4.28)

AIC = 238.13

Smokers without Emphysema are Baseline

The full models contained Age, Smoking duration (year), Pack-Years, FEV1/FVC, Cough (Yes vs. No)

Final model: Log-odds of emphysema = 1.07*Age + 1.02*Pack-Years + 0.93* FEV_1_/FVC + 1.91*Cough

### MMP/ TIMP as biomarkers for presence of emphysema

**Fig. 2:**
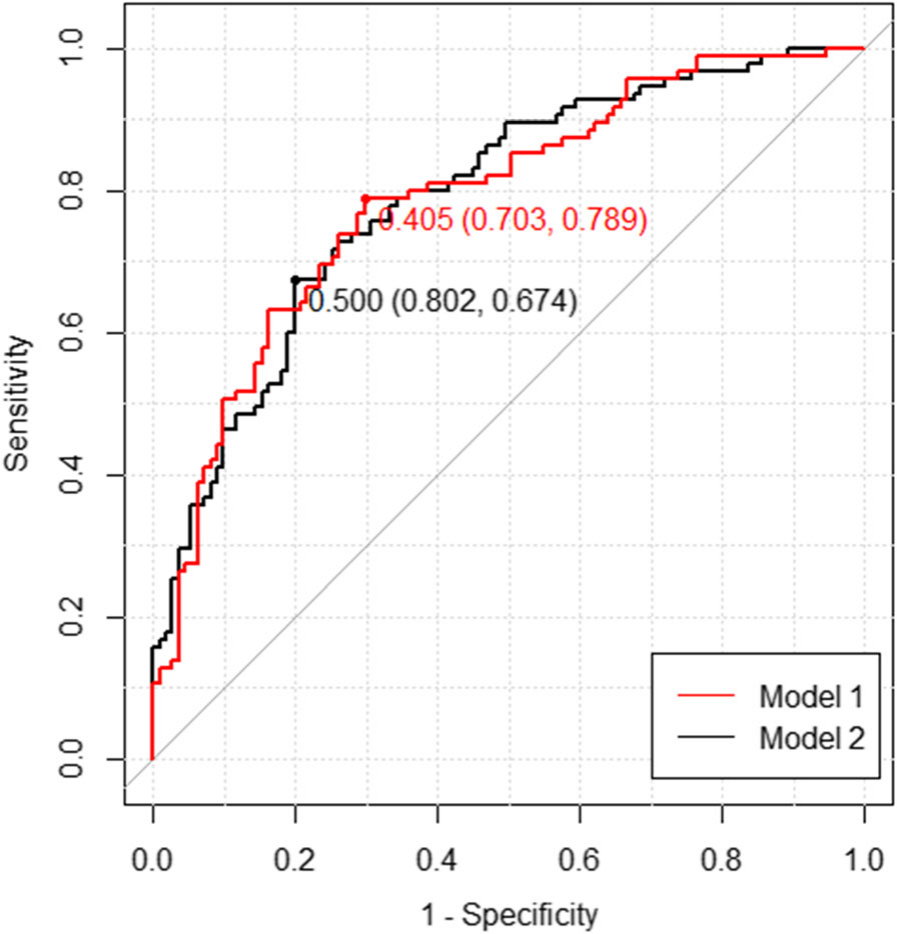
ROC Curves of Model 1 and Model 2, with the corresponding Youden Index (specificity, sensitivity). Optimal cutpoints and (sensitivity, specificity) for that cutpoint shown. The optimal cut point for the logistic score in Model 1 has sensitivity 0.789 and specificity 0.703 for the classification of emphysema . The optimal cut point for the logistic score in Model 2 has sensitivity 0.802 and specificity 0.674

In order to explore the potential ability to predict emphysema using biomarker levels, we added biomarkers log_2_ (TIMP-1), log_2_ (TIMP-2), log_2_ (MMP-1), log_2_ (MMP-2), log_2_ (MMP-7) and log_2_ (MMP-9) and obtained Model 2 (Table 3)**.** Age, FEV1/FVC and pack-years remained statistically significant, but none of the biomarkers showed a significant association with emphysema. We further compared the AIC of these models. The AIC of Model 1 is 238.1 and the AIC of Model 2 is 237.93 with the addition of the biomarkers as predictors. The ROC curves for Models 1 and 2 are shown in Fig. 2, with AUC as 0.788 and 0.789 respectively. The optimal cut-point based on the Youden Index for Model 1 is 0.405 (sensitivity 0.789 and specificity 0.703), and for Model 2 is 0.500 (sensitivity 0.674 and specificity 0.802) (Fig. 2). These results suggest that the serum-based MMP biomarkers do not add additional value to clinical characteristics predicting emphysema in smokers.

**Table 3:** Odds Ratios and 95% Confidence Interval (CI) for Emphysema Based on Stepwise Multiple Logistic Regression models with Clinical Characters and Biomarkers. (222 subjects)

Predictors	OR	95% CI
Age	1.06	(1.01,1.11)
FEV_1_/FVC	0.92	(0.88,0.96)
Pack-Years	1.02	(1.01,1.04)
log_2_(MMP-1)	0.75	(0.52,1.06)
log_2_(MMP-7)	1.40	(0.98,2.02)

AIC = 237.93

Smokers without Emphysema are Baseline

The full models contained Age, Smoking duration (year), Pack-Years, FEV1/FVC, Cough (Yes vs. No), log_2_(TIMP-1), log_2_(TIMP-2), log_2_(MMP-1), log_2_(MMP-2), log_2_ (MMP-7), log_2_(MMP-9)

Final model: Log-odds of emphysema = 1.06*Age+ 1.02*Pack-Years+ 0.92* FEV_1_/FVC + 0.75 *log_2_(MMP-1) + 1.40* log_2_(MMP-7)

## Discussion

In our cohort of lung cancer screening subjects, low dose CT scans were able to detect a high percentage of radiographic emphysema (46%); in addition, spirometry detected significant airway obstruction among heavy smokers who had minimal respiratory symptoms. These results confirm that many smokers are likely underdiagnosed for COPD, and there may be a role in screening certain smokers for COPD. This study showed that serum-based MMP biomarkers did not improve prediction of emphysema in smokers but that traditional clinical information such as age, lung function, and smoking history were better predictors.

The Global Initiative for Chronic Obstructive Lung Disease requires spirometry for the clinical diagnosis of COPD and recommends an assessment of symptoms, the severity of airflow limitation, history of exacerbations, and comorbidities [11]. Spirometry cannot fully categorize the heterogeneity in COPD, but concomitant CT-scans can evaluate the extent of emphysema and other findings such as airway remodeling, gas trapping, and regional ventilation abnormalities [12]. The COPD Gene study enrolled 10,131 study subjects with > 10 pack-years and found increasing emphysema scores with mild COPD (4.4 adjusted mean) to moderate (5.3) to severe emphysema (11.0) [13].

Matrix metalloproteinases and their tissue inhibitors are involved in matrix dissolution and remodeling which makes them reasonable targets for evaluation in emphysema, a destructive disease of the lung matrix. The TIMPs are produced by macrophages and epithelial cells, and the N-terminal domains inhibit all MMPs by binding to their catalytic domain. Serum levels of MMPs -1, -2, -7, -9, and TIMP-1 have been reported to be elevated in emphysema subjects and correlate with the GOLD stages [14]. D’Armiento and colleagues studied severe emphysema using bronchoalveolar lavage fluid and found increased MMPs -1, -9, -12, collagenase and elastase activity in emphysema subjects compared to non-smoking controls. There was a decrease in plasma MMP-1, -9, and TIMP-1 in emphysema compared to controls and no association with FEV_1_/FVC percent predicted or change in FEV_1_ over 3, 6, or 18 months of follow-up. The same investigators also studied MMP-1 transgenic mice and found morphological and physiological evidence for emphysema in this model expressing the human MMP-1 gene [15]. Cigarette smoke targets the MMP-1 promoter and stimulates the extracellular regulated kinase/mitogen-activated protein kinase pathway to degrade types I/III collagens in the lung [16, 17]. However, in our study, we were not able to identify a biomarker for emphysema based on serum MMP levels.

Alcaide and colleagues studied 203 subjects with low-dose CT scans in a lung cancer screening program and found that visually detected emphysema (*n* = 154) was associated with quality of life impairment, an abnormal diffusing capacity, and a significant drop in SpO_2_ during the 6-min walking test [18]. In those with emphysema with DLCO< 80% (*n* = 66) compared to those with emphysema and DLCO> 80% (*n* = 73), there was a significantly reduced FEV_1_ percent predicted (96 ± 15% versus 105 ± 16%; *p* < 0.01). We also found a decrease in the FEV_1_/FVC in those with visual emphysema on their CT-scans.

One of the limitations of this study is that this is a single center observational study with a small number of subjects. Severity, frequency, and pattern of emphysema were not assessed to evaluate whether these features are associated with MMP levels. Detection of emphysema can be delayed because it may take up to 30% of lung destruction before there is airflow limitation. Importantly, we should continue to use clinical predictors such as age, smoking history, and pulmonary function to screen those at risk of developing COPD as there is no benefit to the addition of serum MMP biomarker.

## Supplementary information

**Additional file 1: Supplement Table 1.** Processing Batch by Emphysema Status in Smokers (222 subjects). **Supplement Table 2.** Batch Comparisons for Emphysema and Non-Emphysema: *P*-value: Wilcoxon rank sum test before and after standardization. **Supplement Figure 1.** Box plots for the logarithm to the base 2 of TIMP-1, -2 and bead immunoassay for Matrix Metalloproteinases-1, -2, -7, -9 by Emphysema Status in Smokers in Batch 1 and Batch 2. (222 subjects).

## Data Availability

All data generated or analysed during this study are included in this published article [and its supplementary information files]. The datasets during and/or analysed during the current study available from the corresponding author on reasonable request.
