# Supplementary material for: Value of metalloproteinases in predicting COPD in heavy urban smokers
Source: Respir Res. 2020 Sep 2;21:228. doi: 10.1186/s12931-020-01496-9 (PMC7465798; doi:10.1186/s12931-020-01496-9)
Supplement: Supplementary file 1 — Additional file 1: Supplement Table 1. Processing Batch by Emphysema Status in Smokers (222 subjects). Supplement Table 2. Batch Comparisons for Emphysema and Non-Emphysema: P-value: Wilcoxon rank sum test before and after standardization. Supplement Figure 1. Box plots for the logarithm to the base 2 of TIMP-1, -2 and bead immunoassay for Matrix Metalloproteinases-1, -2, -7, -9 by Emphysema Status in Smokers in Batch 1 and Batch 2. (222 subjects). [file 12931_2020_1496_MOESM1_ESM.docx]

**Supplement:**

**Supplement Table**

Supplement 1: Processing Batch by Emphysema Status in Smokers (222 subjects)

| Batch ID^†^ | Smokers without Emphysema | | Smokers with Emphysema | P value^§^ |  |  |
| --- | --- | --- | --- | --- | --- | --- |
| 1 | 68 (56%) | 44 (44%) | | 0.08 |  |  |
| 2 | 53 (44%) | 57 (56%) | |  |  |  |
| ^§^ Fisher’s exact test  bolded numbers are significant (p≤0.05) | | | |  | |  |

**Supplement 2: Batch Comparisons for Emphysema and Non-Emphysema: P-value: Wilcoxon rank sum test before and after standardization**

|  | Before Standardization | | After Standardization | |
| --- | --- | --- | --- | --- |
|  | **Emphysema** | **Non-Emphysema** | **Emphysema** | **Non-Emphysema** |
| TIMP1 | **2.948e-06** | **0.001007** | 0.3059 | 0.3988 |
| TIMP2 | **0.02024** | **0.002806** | 0.9973 | 0.6142 |
| MMP1 | 0.7039 | 0.4456 | 0.4492 | 0.6779 |
| MMP2 | **1.009e-07** | **8.768e-12** | 0.7762 | 0.5222 |
| MMP7 | **1.839e-06** | **5.728e-13** | 0.4616 | 0.2326 |
| MMP9 | **0.0001543** | **0.01491** | 0.3678 | 0.08806 |

**Supplement Figure**

**Supplement Figure 1: Box plots** for the logarithm to the base 2 of TIMP-1, -2 and bead immunoassay for Matrix Metalloproteinases-1, -2,-7,-9 by Emphysema Status in Smokers in Batch 1 and Batch 2. (222 subjects)


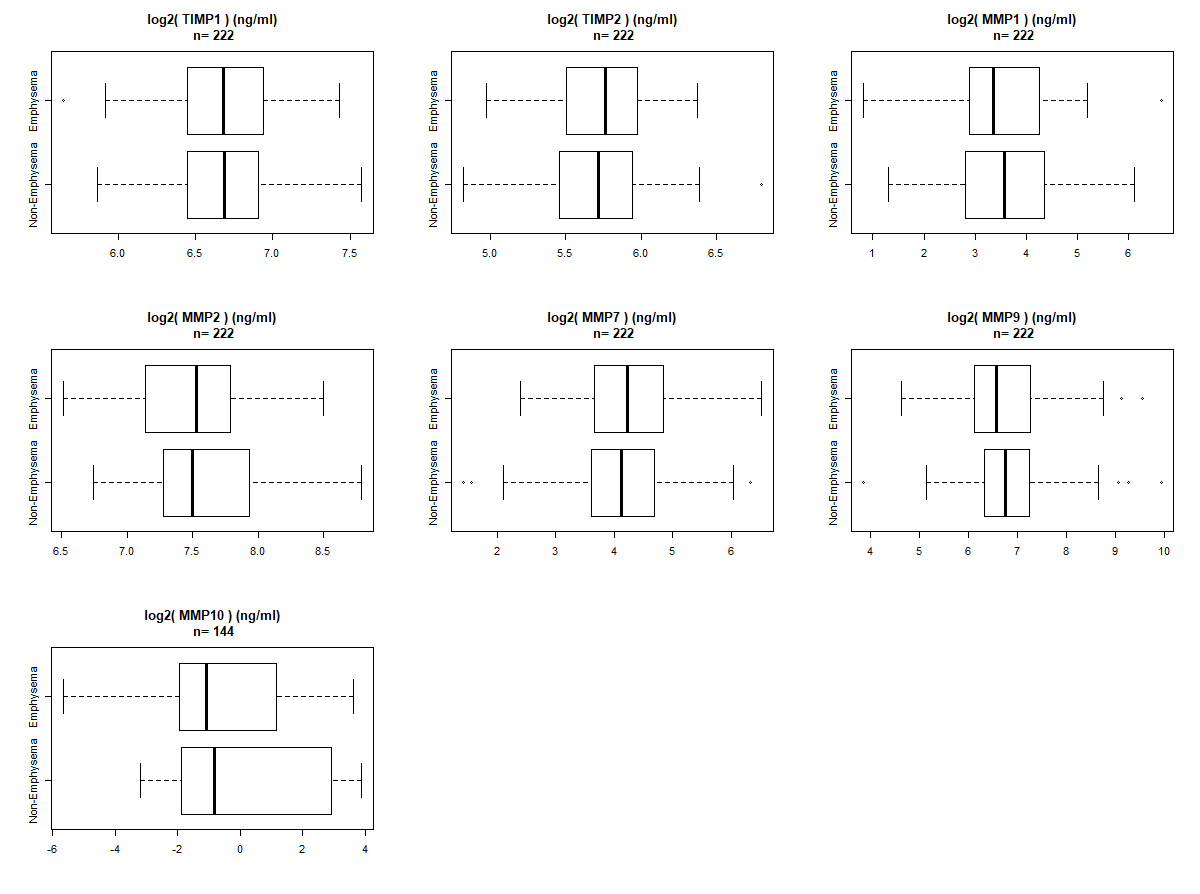


**Supplement Figure Legends**

**Supplement Figure 1: Box plots** for the logarithm to the base 2 of TIMP-1, -2 and bead immunoassay for Matrix Metalloproteinases-1, -2,-7,-9 by Emphysema Status in Smokers in Batch 1 and Batch 2. (222 subjects)
